# Socio-Ecological Risk Factors for Prime-Age Adult Death in Two Coastal Areas of Vietnam

**DOI:** 10.1371/journal.pone.0089780

**Published:** 2014-02-26

**Authors:** Deok Ryun Kim, Mohammad Ali, Vu Dinh Thiem, Thomas F. Wierzba

**Affiliations:** 1 International Vaccine Institute, SNU Research Park, Nakseongdae-dong, Gwanak-gu, Seoul, Korea; 2 Johns Hopkins Bloomberg School of Public Health, Baltimore, Maryland, United States of America; 3 National Institute of Hygiene and Epidemiology, Hanoi, Vietnam; Kenya Medical Research Institute (KEMRI), Kenya

## Abstract

**Background:**

Hierarchical spatial models enable the geographic and ecological analysis of health data thereby providing useful information for designing effective health interventions. In this study, we used a Bayesian hierarchical spatial model to evaluate mortality data in Vietnam. The model enabled identification of socio-ecological risk factors and generation of risk maps to better understand the causes and geographic implications of prime-age (15 to less than 45 years) adult death.

**Methods and Findings:**

The study was conducted in two sites: Nha Trang and Hue in Vietnam. The study areas were split into 500×500 meter cells to define neighborhoods. We first extracted socio-demographic data from population databases of the two sites, and then aggregated the data by neighborhood. We used spatial hierarchical model that borrows strength from neighbors for evaluating risk factors and for creating spatially smoothed risk map after adjusting for neighborhood level covariates. The Markov chain Monte Carlo procedure was used to estimate the parameters. Male mortality was more than twice the female mortality. The rates also varied by age and sex. The most frequent cause of mortality was traffic accidents and drowning for men and traffic accidents and suicide for women. Lower education of household heads in the neighborhood was an important risk factor for increased mortality. The mortality was highly variable in space and the socio-ecological risk factors are sensitive to study site and sex.

**Conclusion:**

Our study suggests that lower education of the household head is an important predictor for prime age adult mortality. Variability in socio-ecological risk factors and in risk areas by sex make it challenging to design appropriate intervention strategies aimed at decreasing prime-age adult deaths in Vietnam.

## Introduction

Understanding the distribution of deaths in space can help public health professionals to quickly analyze spatial relationships and risk factors to facilitate health policy planning and implementation [1]. There is, however, a dearth of knowledge on risk factors and spatial variation of risk for prime-age (15 to <45 years old) adult persons in developing countries. This situation persists despite knowing that a death in this age group profoundly impacts family who experience intangible losses such as diminished guidance, care and companionship, and tangible losses like diminished household income [2]. Unfortunately, observations of infant and child mortality including the risks, causes, and mortality trends cannot be extrapolated to adults [3].

Mortality risk may vary geographically due to changing patterns of risk and differential access to healthcare [1]. Thus, it is important to understand the geographic distribution of mortality so that area-based intervention strategies can be planned. Mapping of raw mortality rates is considered inappropriate as it does not account for the spatial heterogeneity of the population at risk. Although older frequentist methods of analysis are considered appropriate, the newer but less well known Bayesian approach based on Markov chain Monte Carlo (MCMC) is gaining importance [4].

In Vietnam, the national census of Vietnam provides some general mortality data [5]. However, the mortality data and research in the prime-age group is relatively rare. Byass et al. [6] have previously estimated a mortality rate by age and sex from a sample drawn from the population of one district in northern Vietnam. Ngo et al. [7] evaluated total and cause-specific mortality by sex in three broader age groups from a national verbal autopsy survey conducted for the first time in Vietnam. However, those data were not used to investigate risk factors or spatial variation of mortality risk. In this paper, we used a multiple-membership-multiple-classification (MMMC) model [7], a Bayesian hierarchical spatial model that borrows strength from neighbors, to evaluate socio-ecological risk factors and to create spatially smoothed risk maps for prime-age adult death in two coastal areas of Vietnam.

## Methods

### Study area

The study was conducted in two cities of Vietnam: Nha Trang and Hue. Nha Trang is the provincial capital of Khanh Hoa Province. The study area of Nha Trang included 16 out of 26 communes encompassing 150 km^2^. It is transversed by two major rivers: Cai and Lo. Hue City, consisting of 25 communes, is one of the nine districts of Thua Thien that encompasses 72 km^2^. The city is transversed by the Perfume River. Motorbikes are the main mode of transport in both areas. Both areas are frequently visited by Vietnamese and international tourists. The sites were chosen as there were reliable population databases that had been updated yearly by collecting vital demographic events.

### Demographic surveillance and geographic information system

A census was conducted at the beginning of the project by project staff. Subsequently, vital demographic events including births, deaths and migrations of the study population were collected annually to update the population database, TYVTN and SHVTN for Hue and Nha Trang, respectively. During the study period, 170,488 people lived in Nha Trang (2001–2002) and 240,394 people in Hue (2002–2003). In Hue, verbal autopsies were conducted. This recorded information was reviewed by one medically trained coder to define the cause of death according to locally adapted classification of diseases. In Nha Trang, the data on the causes of death were abstracted from hospital records that were coded according to the WHO ICD-10 classification scheme. A global positioning system (GPS) survey was conducted in 2003 to record the geographic coordinates of the residences of all the households in the study areas. Households sharing a single structure or closely connected structures (e.g. multistory buildings) were referenced by a single point. A total of 43,148 households were referenced by 13,566 points in Nha Trang, and 58,141 households were referenced by 11,235 points in Hue.

### Neighborhood definition and data aggregation

**Figure 1:**
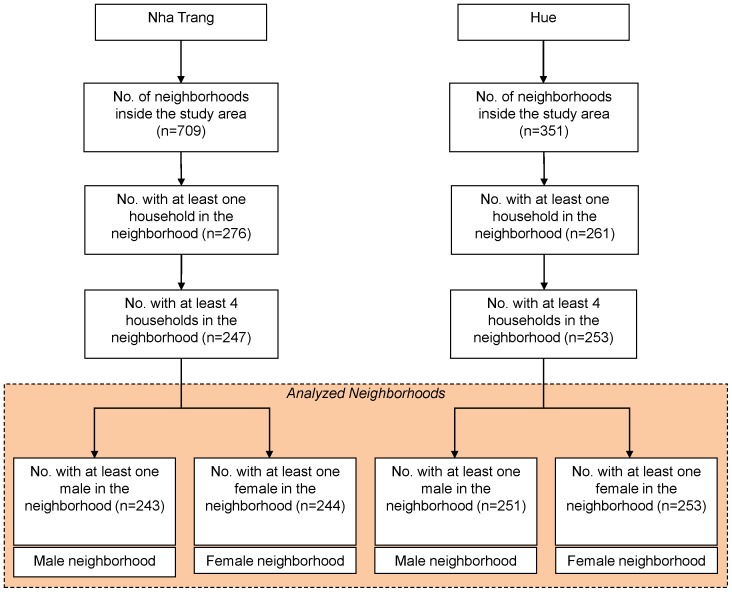
Flow of assembling the neighborhoods for analysis.

The study areas were divided into 500×500 meter cells to define a neighborhood, yielding 276 and 261 neighborhoods with at least one household in Nha Trang and Hue, respectively. We excluded neighborhoods with fewer than four households because ecological data derived from few observations could bias the outcome. We defined the neighborhood by sex with at least one occupant of the respective sex group, and accordingly 243 and 244 neighborhoods were obtained for male and female, respectively, in Nha Trang. In Hue, we obtained 251 and 253 neighborhoods for male and female, respectively. The flow of assembling the neighborhoods for analysis is shown in Figure 1.

Population data and deaths during 2001–2002 from Nha Trang and 2002–2003 from Hue were extracted from the study database and were aggregated by the neighborhoods defined above. For the analysis, we appraised several variables that were previously found to be associated with higher mortality, which included: poor literacy [8]–[11], use of unsafe water for drinking [12], distance to the health care center (hospital/polyclinic) [11], and household headship [13]. We also selected distance to the river from household, as both areas were witnessing many drowning events, particularly among inhabitants 15 to 35 years of age [14]. The neighborhood level variables were derived by summarizing the characteristics of the individuals in the neighborhood; thus they provided information on true neighborhood level constructs [15]

The spatial analysis methods used in this study are as follows:

### Spatial Autocorrelation (Moran's I)

The *Moran's I* statistic is similar to the Pearson correlation coefficient [16] and was calculated as follows: 
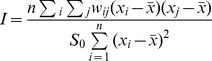
where *n* is the number of neighborhoods, *w_ij_* is the element in the spatial weights matrix corresponding to the observation pair *i, j*. *x_i_* and *x_j_* are observations for neighborhoods *i* and *j* with mean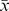. 
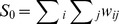
The weight was defined as 
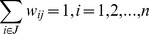
where *j* is the set of neighbors contiguous to neighborhood *i*. The *Moran's I* was computed using GeoDa 0.9.5-i5 software [17]. A significance test against the null hypothesis of no spatial autocorrelation through a permutation procedure of 9999 Monte Carlo replications was used to test for the significance of the statistic.

### Multiple-membership-multiple-classification (MMMC) model

We used a MMMC model, a Bayesian hierarchical spatial model, which considers observed counts for a set of areas with a known neighborhood structure, and uses Bayesian method in conjunction with suitable prior distribution using Markov chain Monte Carlo procedures to estimate fixed effects, and two sets of neighborhood random effects (unstructured effects *u_j_* of variance 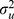 and spatially structured spatially correlated random effects *s_j_* of variance 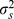) [18]–[19]. Here, *u_j_* takes independent values in each neighborhood and therefore captures unstructured neighborhood variations, which assumes neighborhood variations close to each other are not similar; *s_j_* adopts more similar values for neighborhoods close to each other, thereby by reflecting spatially structured variations. The structured part allowed us to borrow strength from neighbors to make the estimates more robust. We chose the 1^st^ order of neighbor based on overall assessment of the Deviation Information Criterion (DIC) value of the different orders of neighbor [20]. We computed the proportion of total neighborhood variance that is spatially structured as 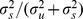. To complete Bayesian specification in the model, we set uniform prior for fixed effect and inverse Gamma (0.001, 0.001) prior for a precision parameter of random effects. The estimates are based on a total sample size of 50,000 after burn-in of 20,000 iterations.

We used MLwiN version 2.1 [21] to analyze the data. The MLwiN offers several choices of priors for the model parameters and in particular for variance parameters, it offers two sets of diffuse priors. The default is the inverse gamma priors but a uniform prior for random effects is also offered. We chose the default diffuse priors since the Bayesian approach based on diffuse prior does not yield significantly different portfolio decisions compared with the classical framework [22].

We developed the covariate model and computed spatially smoothed relative risk (SSRR) for mortality derived from the model, classified in four groups at 0.50 intervals of risk for better interpretation of the outcomes, and mapped the SSRR of male and female mortality of both the study sites. The classification of SSRR = 1.01–1.50 was treated as moderate risk and SSRR≥1.51 as high risk.

### Ethics

The study in Hue, Vietnam was approved by the Institutional Review Board of the International Vaccine Institute (IVI), WHO Secretariat Committee on Research Involving Human Subjects (SCRIHS), WHO, Geneva, and the Ethical Review Board of the National Institute of Hygiene and Epidemiology (NIHE), Hanoi, Vietnam. The study in Nha Trang, Vietnam was approved by the WHO Secretariat Committee on Research Involving Human Subjects (SCRIHS), and the Ethical Review Board of the National Institute of Hygiene, Hanoi, Vietnam. The study databases were anonymized to maintain subject's confidentiality. In Nha Trang, verbal informed consent was taken while registering the population, but written informed consent was taken while recruiting the cases in the study. In Hue, both community and individual consent were obtained from the community leaders and the participants (or the parents/guardians). Because communities rather than individuals were allocated in the study interventions, verbal informed consent was taken as accepted by the local ethics committees. Besides, special approval was taken from the local education authorities. In case of specimen collection, individuals written informed consent were obtained.

## Results

We used routinely collected population and vital demographic events data for the spatial models. There were 93,039 prime-age adults in Nha Trang and 130,837 adults in Hue. Among these individuals, 199 deaths occurred in Nha Trang and 333 deaths in Hue during the study period. Annual prime-age mortality rates were 1.64/1000 and 0.54/1000 for males and females, respectively, in Nha Trang, and 1.82/1000 and 0.74/1000 for males and females, respectively, in Hue.

**Figure 2:**
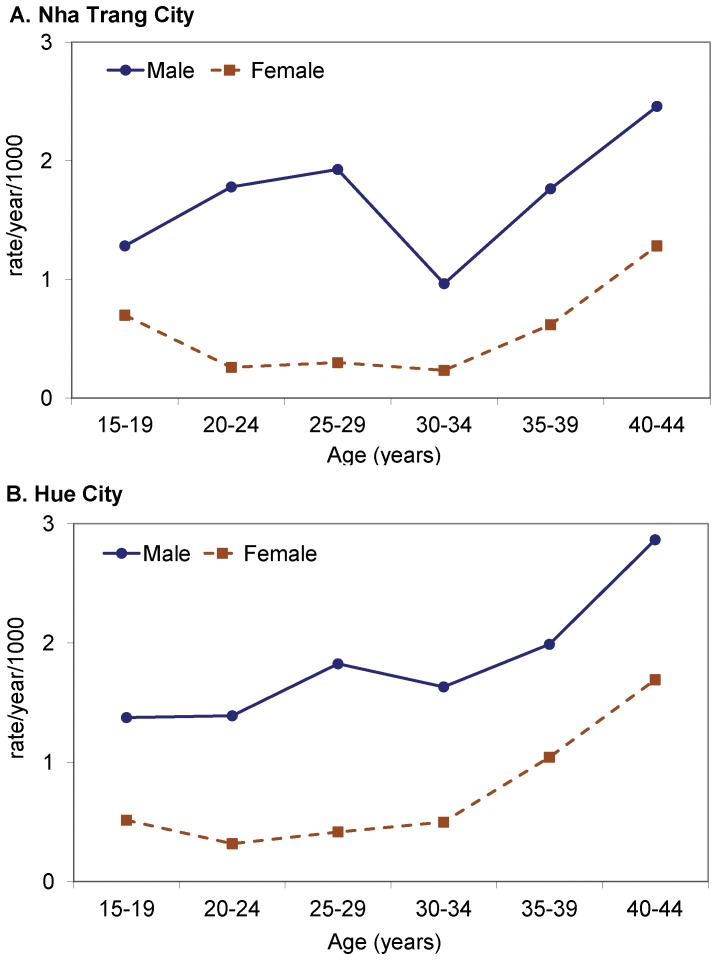
Prime-age adult mortality rate by age and gender in the Nha Trang and Hue cities of Vietnam.

We used the death records from the hospitals in Nha Trang and the verbal autopsies of deaths in Hue to determine the causes of death. Note that not all deaths were recorded in Nha Trang, and we could not collect the verbal autopsies of all deaths in Hue during the study period. Among the data on the causes of deaths, traffic accidents, drowning, and cardiovascular diseases were the first, second, and third leading causes of death for males in the two coastal areas of Vietnam (Table 1). Traffic accident was the first leading cause of death for females followed by both suicide and sudden death from unknown causes. Mortality rates were observed to vary by age and sex among prime-age adults (Figures 2a and 2b). In general, mortality rates increased with age.

**Table 1:** Causes of death among prime-age adults in the two cities of Vietnam.

Causes of death	Male	Female	Total
	N (%)	N (%)	N (%)
Traffic accident	42 (45)	7 (30)	49 (43)
Drowning	11 (11)	1 (4)	12 (10)
Cardiovascular disease (CVD)	6 (6)	2 (8)	8 (7)
Suicide	3 (3)	4 (17)	7 (6)
Cancer	4 (4)	3 (13)	7 (6)
Others (Somnolence, stupor, coma, Asphyxiation, Shock, Severe burning, homicide)	5 (5)	1 (4)	6 (5)
Fall from height	5 (5)	0 (0)	5 (4)
Sudden death, unknown cause	1 (1)	4 (17)	5 (4)
Respiratory disease	4 (4)	1 (4)	5 (4)
Hepatic failure	3 (3)	0 (0)	3 (3)
Intracranial injury	3 (3)	0 (0)	3 (3)
Food poisoning	2 (2)	0 (0)	1 (1)
Diabetes mellitus	1 (1)	0 (0)	1 (1)
Inflammatory polyneuropathy	1 (1)	0 (0)	1 (1)
Intracerebral haemorrhage	1 (1)	0 (0)	1 (1)
Tetanus	1 (1)	0 (0)	1 (1)
Total	92 (100)	23 (100)	115 (100)

Note: The causes of 115 out of 532 deaths were collected from the hospital records in Nha Trang, and from a verbal autopsy study in Hue.

The results of the *Moran's I* using the 1^st^ order of neighbor yielded significant clustering of mortality in all groups, except for females in Nha Trang (in Nha Trang, *Moran's I* for male = 0.21, p<0.01 and for female = 0.03, p = 0.2; In Hue, *Moran's I* for male = 0.36, p<0.01 and for female = 0.14, p<0.01). The null model shows 64% neighborhood variability was spatially structured for males and 61% for females in Nha Trang, which were 69% and 84% for males and females, respectively, in Hue (Tables 2 and 3).

**Table 2:** Model estimates for prime-age adult mortality in Nha Trang, Vietnam.

Parameters	Null model			Covariate Model		
	Estimates	SE	P-value	Estimates	SE	P-value
*Men*						
Fixed effect						
Intercept	-0.053	0.103	0.608	1.884	0.906	0.037
Percent of male household head	—	—	—	−0.021	0.012	0.092
Percent of household head having at least 5 years of schooling				−0.008	0.005	0.108
Random effect						
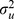 (unstructured component)	0.065	0.084	0.436	0.052	0.071	0.469
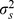 (structured component)	0.114	0.253	0.652	0.027	0.054	0.622
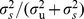	0.64			0.34		
DIC	372			372		
*Women*						
Fixed effect						
Intercept	−0.062	0.175	0.724	0.707	0.298	0.018
Percent using tap water	—	—	—	−0.009	0.004	0.019
Average distance from household to river (km)				−0.089	0.045	0.049
Random effect						
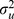 (unstructured component)	0.131	0.244	0.592	0.147	0.172	0.394
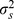 (structured component)	0.207	0.316	0.313	0.175	0.332	0.597
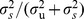	0.61			0.54		
DIC	236			229		

SE: standard error; DIC: Deviance Information Criterion.

**Table 3:** Model estimates for prime-age adult mortality in Hue City, Vietnam.

Parameters	Null model			Covariate Model		
	Estimates	SE	P-value	Estimates	SE	P-value
*Men*						
Fixed effect						
Intercept	−0.053	0.082	0.519	1.006	0.288	<0.001
Percent of household head having at least 5 years of schooling	—	—	—	−0.014	0.004	0.001
Average distance from household to river (km)	—	—	—	−0.042	0.019	0.026
Random effect						
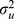 (unstructured component)	0.054	0.053	0.309	0.027	0.034	0.419
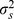 (structured component)	0.118	0.182	0.519	0.101	0.192	0.600
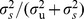	0.69			0.79		
DIC	477			474		
*Women*						
Fixed effect						
Intercept	−0.023	0.110	0.834	−0.249	0.185	0.133
Average distance from household to healthcare (km)	—	—	—	0.021	0.012	0.073
Random effect						
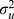 (unstructured component)	0.034	0.054	0.532	0.040	0.061	0.513
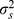 (structured component)	0.184	0.339	0.587	0.047	0.091	0.606
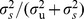	0.84			0.54		
DIC	342			341		

SE: standard error; DIC: Deviance Information Criterion.

The results of the covariate model (created with only with those variables that show significant at p<.10 in a bivariate model) for males in Nha Trang showed that none of the variables were significantly associated with mortality in this group (Table 2). A lower percentage of using tap water (normally a safe water source) for drinking in the community and a shorter distance between the household and a river were significant factors associated with higher female mortality in Nha Trang (Table 2). On the other hand, lower percent of literate household in the community and shorter distance from household to river had significantly higher male mortality in Hue. None of the variables had any significant association with female mortality in Hue (Table 3). Except for males in Hue, the spatial structure neighborhood variability was lower in the covariate model than that in the null model.

**Figure 3:**
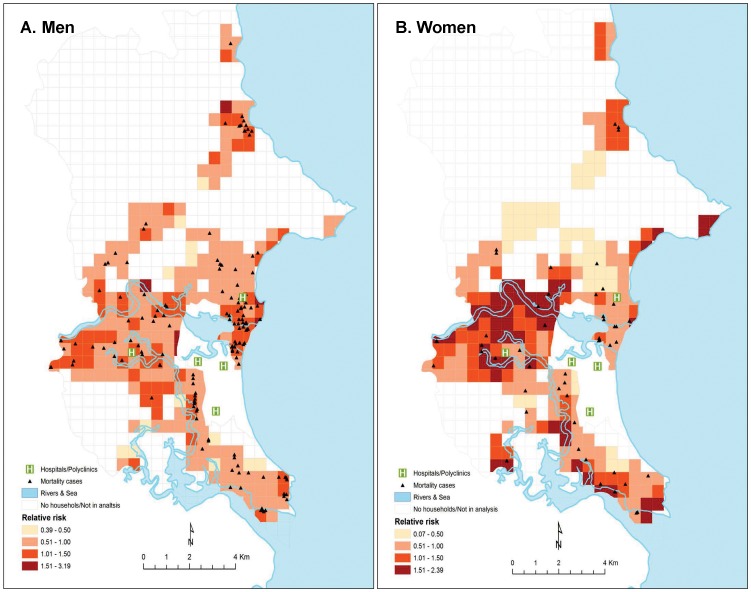
Spatial variation of risk for prime-age adult men and women mortality in a covariate model, Nha Trang, Vietnam, 2001-2002.

**Figure 4:**
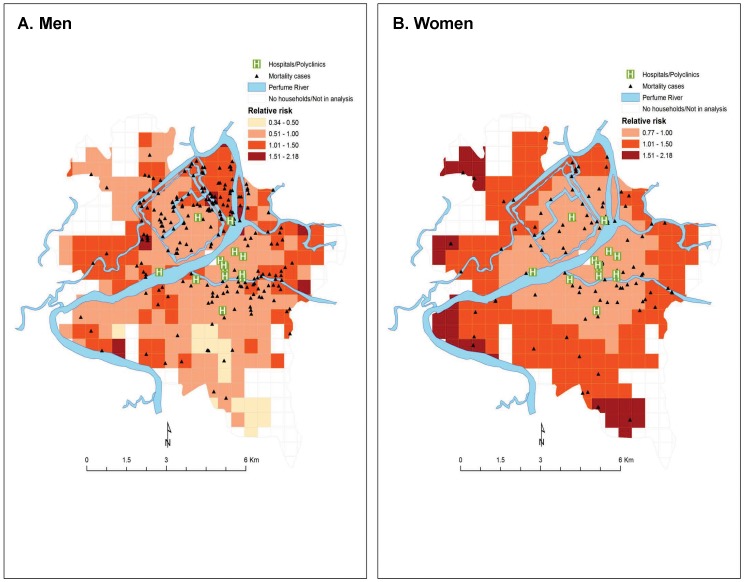
Spatial variation of risk for prime-age adult men and women mortality in a covariate model, Hue City, Vietnam, 2002-2003.

The SSRR for mortality derived from the covariate model, classified in four groups at 0.50 intervals of risk for better interpretation of data, are shown in figures 3 and 4. The SSRR for mortality derived from the model yielded several risk neighborhoods characterized by: a lower percentage of male household heads in the community, a greater percentage using unsafe water for drinking, a lower number of literate households in the community, living in close proximity to a river, and living far from health care center.

## Discussion

We observed that male mortality is higher than female mortality. This observation is consistent with global findings [1], [23]. One of the consequences of the observed male mortality is that it may increase the number of poor female-headed households due to diminished household income [24]. Traffic accidents were the leading cause of male mortality in our study area. The Vietnamese government also reported the primary causes of deaths in this age-group are suicide and accidents [6]. Drowning was the second leading cause of adult male mortality in Vietnam, which could be due to men spending more time swimming. Alcohol consumption among young men may be a contributing factor in traffic-associated and drowning deaths [25]–[26]. Alcohol consumption is very popular during special occasions like birthdays and weddings. Educational materials depicting Vietnamese celebrations should provide alternatives to alcoholic drinks and also encourage people to drink in moderation if they must drink [27].

Cardiovascular disease (CVD) is another major cause of death in this population. Tobacco use is a concern for the Vietnamese community [28] suggesting interventions are needed for tobacco cessation and education on the harmful effects of tobacco smoke on cardiovascular health. Excessive alcohol consumption may also be a contributory factor for CVD. There are also several other CVD risk factors, often clustered within individuals, that are common in the adult population of Vietnam [29]. Combination of population and individual approaches are required to reduce the burden of CVD risk factors and maximize the protective effects for the whole community.

Female suicide was more frequent than male suicide. Similar observations have been made elsewhere [30]. Suicide is stigmatized in Vietnam and is likely to be underreported. Some of the sudden/unknown deaths could be suicides [31].

Communities with a higher proportion of illiterate household heads had significantly higher male mortality in Hue. Our data suggests that having household heads that can read and write is important to preventing deaths [32]–[33]. Given that the measured effects of education are large, investments in education may be cost-effective for preventing these deaths [34]. In contrast, prediction and prevention of suicide is one of the most difficult problems facing mental health professionals [35]. Several studies have demonstrated that an individual's ability to forecast his or her future behavior, such as whether he or she will commit suicide, is often inaccurate [36]–[37]. Still, knowledge of risk factors for suicide need to be translated into practical interventions that can be included in population-based programs addressing depression, mental health, and that integrate and enhance community and primary care for the prevention of suicide [38]. Research on suicide methods is also important for preventing suicidal death [39]–[40].

Living in close proximity to a river was found to be significantly associated with higher male mortality in Hue. This could be the reason why we observed drowning as the second leading cause for male death in Vietnam. Higher female mortality in Nha Trang for persons living in close proximity to a river may be attributed to suicide. Importantly, because suicide is stigmatized in Vietnam, some suicidal cases may have been reported as drowning cases.

The absence of safe water is a major underling cause of death in many parts of the world, and access to potable water is economically constrained in many subsistence economies like Vietnam [12]. The provision of safe, effective, and affordable vaccines against enteric diseases may mitigate the risk of death related to poor sanitation in some areas [41]. We observe that use of unsafe drinking water was associated with higher female mortality in Nha Trang. In the study area, people use diverse sources of water, such as, tap, well, hand pump, water vendor, and river. Although we do not expect intra-household difference in water use behavior, little is known about how water is allocated among household members [42]. Consumption rates indicate that, on average, women consume more water in the households than men [42]. Even where tap water use is common, variation in use and, therefore, illness can be affected by water use practices [43]. The results of our study suggest there could be intra-household differences in water use practices and consumption in the study area.

In our study, we used a spatial hierarchical approach to analyze the data and to create the risk maps for the prime-age adult deaths. The results of the Moran's I test indicated that a hierarchical spatial model is an appropriate method for these data sets. Usually, a hierarchical model does not incorporate notion of space, i.e. spatial connection between neighborhoods is ignored. Therefore, measures of variations in such models provide only partial insight into the spatial distribution of outcome [44]. Considering both the structural variance and the spatially unstructured component, one can understand which areas deviate from the latent structure. Our models show that higher neighborhood level variation is spatially structured, thus coordinating interventions between neighborhoods may be an effective public health strategy. Additionally, the reduction of neighborhood level variability in the covariate model from the null model suggests that the covariate model is well suited for identifying ecological risk factors for deaths. Research on the impact of neighborhood on morbidity and mortality has been conducted using standard multilevel models [45]. In case of less frequent events such as adult mortality, ignoring spatial context while conducting an ecological analysis may be affected by the random variation caused by the instability of observed rates in small neighborhoods. The results of our study suggest a deeper understanding of spatial variations in health outcomes may be gained by building the notion of space into a hierarchical spatial model for measuring contextual factors across continuous space [46].

The potential limitations of our study are the choice of neighborhood size and the variations in the size of population across neighborhoods. We believe our selection is an appropriate compromise between loss of resolution and excess dispersion. Another limitation is that our data on the causes of mortality from Nha Trang was collected from hospital records, and is not population based. These deaths were added retrospectively to the database. Furthermore, verbal autopsy for identifying the cause of death in Hue was not done for all deaths in the study area. However, our data are consistent with previous findings [1], [6], [44]; thus we believe that the data used here for determining the causes of death are representative. The national data on the causes of death in Vietnam can be found in appendix. Another limitation is that we did not find any pregnancy related death among females due to paucity of data on the causes of death. Identification of maternal death may also be less frequent due to: inadequate understanding of the aforementioned ICD rule, inadequate completion of death certificates (completed without mention of pregnancy status), the desire to avoid litigation, and the desire to suppress information, especially in case of death due to abortion [47]. This could perhaps contribute to increased number of unknown death among females. The other limitation is that the population database was updated annually using vital demographic events. However, the results of our study suggest such changes in population dynamics did not influence our mortality estimates.

Many health data are collected with a known but complex underlying structure [8]. Often this underlying data structures cannot be fitted into a nested structure. First there are cross-classified models where the classifications in the data are not nested. Secondly we consider multiple membership models where an observation does not belong simply to one member of a classification. These two extensions when combined allow us to fit models to a large array of underlying structures. Existing frequentist approaches to fitting such data have some important computational limitations. In this paper we overcame such limitations using Bayesian methods, since Bayesian model fitting is easily accomplished using Monte Carlo Markov chain (MCMC) techniques.

In conclusion, our study provides the first insight into the spatial distribution of gender-specific mortality in Vietnam and identifies the ecological context for the higher mortality in the prime-age group. The observed differences in the socio-ecological risk factors and the risk areas by sex may make it challenging to devise intervention strategies aimed at decreasing prime-age adult deaths.

## Supporting Information

Table S1 **Proportion of deaths occurring in the 12 months prior to the census by cause of death, sex, urban/rural residence and socio-economic region, 2009.**
(DOCX)
