# Supplementary material for: Socio-Ecological Risk Factors for Prime-Age Adult Death in Two Coastal Areas of Vietnam
Source: PLoS One. 2014 Feb 26;9(2):e89780. doi: 10.1371/journal.pone.0089780 (PMC3935940; doi:10.1371/journal.pone.0089780)
Supplement: Table S1 — Proportion of deaths occurring in the 12 months prior to the census by cause of death, sex, urban/rural residence and socio-economic region, 2009. (DOCX) [file pone.0089780.s001.docx]

Table S1. Proportion of deaths occurring in the 12 months prior to the census by cause of death, sex, urban/rural residence and socio-economic region, 2009

Unit: Percent

| Residence/Socio-economic region | Total | Cause of death: | | | | | |
| --- | --- | --- | --- | --- | --- | --- | --- |
|  |  | Illness | Labour accident | Traffic accident | Other accidents | Other causes | Not specified |
| **OVERALL** |  |  |  |  |  |  |  |
| **Entire country** | **100** | **82.1** | **1.0** | **4.7** | **3.0** | **8.9** | **0.3** |
| Urban | 100 | 83.4 | 0.8 | 4.6 | 2.2 | 8.7 | 0.3 |
| Rural | 100 | 81.6 | 1.1 | 4.7 | 3.2 | 9.0 | 0.3 |
| **Socio-economic region:** |  |  |  |  |  |  |  |
| Northern Midlands and Mountains | 100 | 82.2 | 1.2 | 3.5 | 3.4 | 9.4 | 0.3 |
| Red River Delta | 100 | 81.0 | 1.2 | 4.1 | 2.3 | 10.9 | 0.4 |
| North and South Central Coast | 100 | 80.1 | 1.2 | 5.6 | 3.2 | 9.5 | 0.4 |
| Central Highlands | 100 | 75.9 | 1.2 | 7.7 | 5.4 | 9.3 | 0.4 |
| Southeast | 100 | 83.0 | 0.8 | 5.4 | 2.5 | 8.2 | 0.1 |
| Mekong River Delta | 100 | 86.9 | 0.7 | 3.9 | 2.7 | 5.6 | 0.2 |
| **MALE** |  |  |  |  |  |  |  |
| **Entire country** | **100** | **80.6** | **1.6** | **6.5** | **3.6** | **7.4** | **0.3** |
| Urban | 100 | 82.5 | 1.2 | 6.1 | 2.7 | 7.2 | 0.2 |
| Rural | 100 | 79.9 | 1.7 | 6.6 | 3.9 | 7.5 | 0.3 |
| **Socio-economic region:** |  |  |  |  |  |  |  |
| Northern Midlands and Mountains | 100 | 81.4 | 1.7 | 4.8 | 3.7 | 8.1 | 0.3 |
| Red River Delta | 100 | 80.6 | 1.9 | 5.4 | 2.8 | 9.0 | 0.3 |
| North and South Central Coast | 100 | 78.6 | 1.8 | 7.8 | 3.9 | 7.6 | 0.3 |
| Central Highlands | 100 | 74.6 | 1.8 | 9.9 | 5.9 | 7.4 | 0.5 |
| Southeast | 100 | 81.1 | 1.1 | 7.7 | 3.5 | 6.6 | 0.0 |
| Mekong River Delta | 100 | 84.2 | 1.1 | 5.7 | 3.7 | 5.1 | 0.3 |
| **FEMALE** |  |  |  |  |  |  |  |
| **Entire country** | **100** | **84.1** | **0.3** | **2.1** | **2.0** | **11.1** | **0.4** |
| Urban | 100 | 84.7 | 0.1 | 2.4 | 1.4 | 10.9 | 0.4 |
| Rural | 100 | 83.9 | 0.3 | 2.0 | 2.3 | 11.2 | 0.4 |
| **Socio-economic region:** |  |  |  |  |  |  |  |
| Northern Midlands and Mountains | 100 | 83.5 | 0.4 | 1.3 | 2.8 | 11.6 | 0.4 |
| Red River Delta | 100 | 81.7 | 0.2 | 2.3 | 1.7 | 13.6 | 0.6 |
| North and South Central Coast | 100 | 82.2 | 0.3 | 2.6 | 2.3 | 12.3 | 0.4 |
| Central Highlands | 100 | 78.2 | 0.3 | 3.9 | 4.6 | 12.6 | 0.4 |
| Southeast | 100 | 85.9 | 0.3 | 1.8 | 1.0 | 10.7 | 0.3 |
| Mekong River Delta | 100 | 90.6 | 0.1 | 1.6 | 1.5 | 6.1 | 0.1 |

Source: The 2009 Vietnam Population and Housing Census: Major Findings
